# Mechanical Stress-Induced Defects in Thick a-PbO Layers

**DOI:** 10.3390/ma18091904

**Published:** 2025-04-23

**Authors:** Janos Rado, Amy Stieh, Attila Csík, Sándor Kökényesi, Alla Reznik

**Affiliations:** 1 Physics Department, Lakehead University, Thunder Bay, ON P7B 5E1, Canada; arstieh@lakeheadu.ca (A.S.); areznik@lakeheadu.ca (A.R.); 2 HUN-REN Institute for Nuclear Research, 4032 Debrecen, Hungary; csik.attila@atomki.hu; 3 Department of Electrical and Electronic Engineering, University of Debrecen, 4032 Debrecen, Hungary; kiki@science.unideb.hu; 4 Thunder Bay Regional Health Research Institute, Thunder Bay, ON P7B 7A4, Canada

**Keywords:** lead oxide, X-ray detector, direct conversion, stress-induced defects, crack development, thermal induced crystallization

## Abstract

Amorphous lead oxide (a-PbO) X-ray photoconductors show potential for applications in direct conversion medical imaging detectors within the diagnostic energy range. a-PbO enables large-area deposition at low temperatures and exhibits no signal lag. Low dark current can be maintained through specialized blocking layers, similar to those used in multilayer amorphous selenium (a-Se) structures in commercial detectors. However, the current state of a-PbO technology faces challenges in thick layer deposition, leading to crystalline inclusions and cracks. Our proposed stress-induced crystallization model reveals that intrinsic stress in a-PbO layers amplifies with thickness, leading to crystallographic defects. These defects, which are associated with the stable phase of β-PbO, contribute to increased dark current and initiate layer cracking. We calculate the thermal expansion coefficient of a-PbO, indicating a thermomechanical mismatch between the photoconductor and the substrate as the primary source of stress. Furthermore, we demonstrate that layer deposition parameters significantly impact heat accumulation within the growing layer, thereby facilitating temperature-induced crystallization. Our study suggests that relieving stress in grown a-PbO layers by eliminating thermal expansion coefficient mismatches between different layers in a-PbO blocking structures, coupled with optimizing deposition parameters to prevent heat accumulation during layer growth, may inhibit or even prevent stress-induced crystallization and the emergence of structural defects in thick a-PbO layers.

## 1. Introduction

Large-area flat-panel X-ray imagers (FPXIs) based on amorphous silicon (a-Si:H) digital flat-panel technology have revolutionized diagnostic imaging and image-guided interventional procedures due to their capability for creating high-resolution images and signal readouts at a high frame rate. Commercially available direct conversion FPXIs utilize a uniform layer of stabilized amorphous selenium (a-Se) photoconductor [1,2] deposited over readout electronics employing a two-dimensional array of a-Si:H thin film transistors (TFTs). X-ray photons absorbed by the a-Se generate electron–hole pairs, which are separated by an applied electric field and subsequently read out by the TFT array. Practical a-Se direct conversion detectors incorporate thin blocking layers between the photoconductor and the contacts to prevent charge injection from the contacts and reduce dark current.

While the imaging properties of a-Se-based FPXIs for various medical imaging modalities have been analyzed [3,4], the primary application remains mammography [5], where the average X-ray photon energy is around 20 keV. Indeed, a-Se is efficient only with “soft” X-rays due to its low atomic number (Z) [6,7]. For applications requiring higher photon energies, materials with higher Z and X-ray attenuation coefficients are needed to ensure near-complete absorption within a practical photoconductor thickness (e.g., 100–500 µm).

To this end, high-Z X-ray photoconductors, such as polycrystalline layers of BiI3 [8], PbI2 [9,10], HgI2 [11,12,13], ZnO [14], CdTe [15], Cd1-xZnxTe [16], and PbO [17,18], have been considered as potential a-Se replacements in direct conversion detectors for the diagnostic energy range. The conversion gain of these materials is 3–8 times higher than that of a-Se, which has potential for X-ray quantum noise-limited imaging performance at low exposures, since the X-ray quantum noise can overcome the electronic noise. However, most of these polycrystalline photoconductors to date either suffer from possessing too large a dark current, not having sufficient charge collection efficiency, or not having adequate time response to X-ray irradiation (or from a combination of these problems).

In addition, perovskite semiconductors, which have emerged as some of the most promising materials for optoelectronic applications, have also attracted a great deal of attention for applications in radiation detection [19,20,21]. However, despite recent advancements, perovskite technology is not yet mature enough for commercialization in X-ray detectors [22].

Amorphous Lead Oxide (a-PbO) emerged as a particularly promising alternative to a-Se. a-PbO exhibits two important features: the capability for large-area deposition through ion-assisted thermal evaporation at substrate temperatures not exceeding 100 °C, and the absence of signal lag. The latter refers to the lack of residual current persisting after X-ray exposure, making it particularly suitable for real-time imaging applications like fluoroscopy. Additionally, the dark current of an a-PbO detector can be maintained at an appropriately low level (<10 pA/mm^2^) even under a large electric field (>10 V/µm) by utilizing a thin blocking polymer layer, as recently reported by our group [23].

However, much like other amorphous semiconductors, including non-stabilized a-Se, a-PbO is inherently metastable with respect to its polycrystalline counterpart.

**Figure 1:**
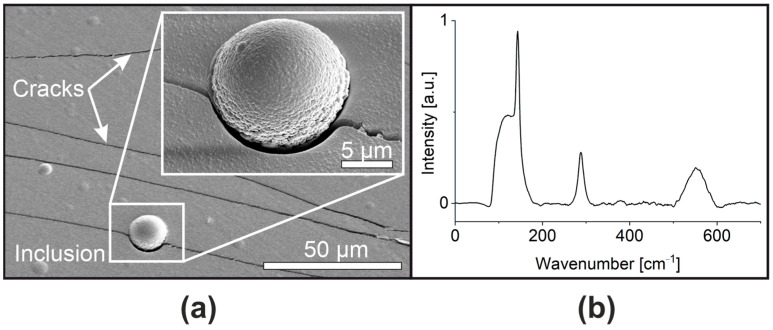
(**a**) SEM image of a 10 μm a-PbO sample showing crystallite inclusion and cracks. (**b**) A Raman spectrum obtained within an inclusion; the peaks at 143 cm^−1^ and 288 cm^−1^ are attributed to orthorhombic PbO [24].

This metastability manifests in a unique way: while thin (<5 µm) a-PbO layers remain stable over an extended five-year observation period, maintaining phase uniformity, thicker layers can undergo structural changes during deposition, leading to the appearance of crystalline inclusions in the grown layer, as demonstrated by cross-section morphological and microstructural characterization with scanning electron microscopy (SEM), and Raman spectroscopy analysis (Figure 1). Optical microscopy revealed sample cracking, with the severity scaling up with the thickness of the a-PbO layer.

We previously hypothesized that, during the ion-assisted physical vapor deposition of a-PbO layers, energy from ion bombardment can cause localized overheating, triggering a structural transformation within the amorphous phase. This effect is pronounced in layers ≥ 10 µm thick, where reduced thermal conductivity limits energy dissipation, leading to localized phase transitions and microcrystallite formation. We further hypothesized that thermal strain between the substrate and a-PbO layer contributes to structural imperfections, including cracking.

Addressing the above challenges in thick a-PbO layer growth requires understanding their root cause and developing deposition conditions to prevent them. This paper tackles both issues through stress analysis in grown layers and proposes a practical approach for creating a-PbO blocking structures (without inclusions and cracks) with extremely low dark currents for X-ray medical imaging applications.

## 2. Materials and Methods

### 2.1. Sample Preparation

Different types of substrates were utilized for distinct measurements of stress in a-PbO layers. To investigate stress- and temperature-induced disorders in thin a-PbO layers, commercially available ITO (indium-tin-oxide) coated boro-aluminosilicate glass substrates were used. For assessing the effect of the blocking layer on stress properties, samples measuring 2.5 cm × 2.5 cm × 0.7 cm were coated with varying thicknesses of polyimide (PI 2610) using the conventional spin-coating technique. The thickness of the PI layers was adjusted simply by altering the speed of the spin coater.

**Figure 2:**
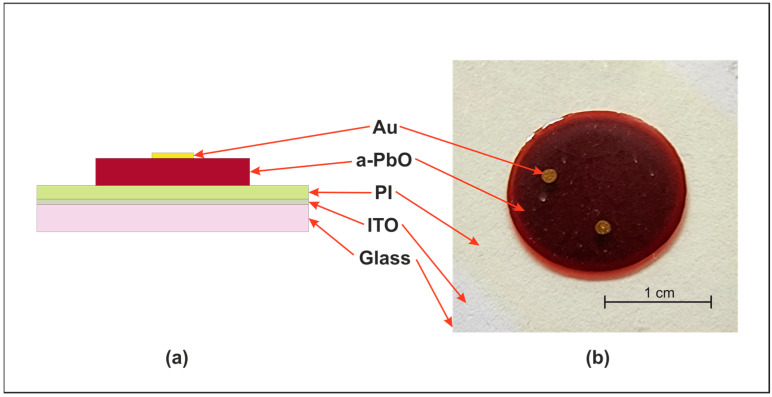
Schematic (**a**) and optical image (**b**) of the PI/a-PbO multilayer detector structure (schematic not to scale).

To calculate the thermal expansion coefficient of a-PbO, we employed substrates with varying thermal properties, including aluminum (6061-T6, al), soda-lime (sl), boro-aluminosilicate (bs), and fused silica (fs). A summary of the substrate types used and their properties is presented in Table 1.

a-PbO layers, with thickness ranging from approximately 2 µm to 25 µm, were deposited using the ion-assisted thermal evaporation technique, as detailed elsewhere [18]. High-purity PbO powder (Chemsavers Inc., Bluefield, WV, USA) was evaporated at ~1000–1100 °C in a background atmosphere of oxygen at a pressure of ~0.1 Pa. The PbO particles condensed on the substrate, while the growing layer was continuously bombarded with oxygen ions. To ensure that the substrate temperature remained below the industry-required maximum of 200 °C, due to the temperature sensitivity of the readout electronics, we continuously monitored the substrate temperature throughout the deposition process. Our measurements confirmed that the substrate temperature never exceeded 150 °C. The energy of the ions remained constant at ~70 eV during all deposition processes, providing a growth rate between 130 and 170 nm/min, depending on the crucible temperature. For ITO-coated substrates, a 150 nm thick Au layer was sputtered onto a-PbO to serve as the electrical top contact (Figure 2).

### 2.2. Experimental Techniques

#### 2.2.1. Stress Measurement

Stress measurements were conducted at 25 °C, using an AlphaStep D-100 profilometer (KLA Corporation, Milpitas, CA, USA). The surface was scanned along the same track with identical settings before and after a-PbO layer depositions. Shadow masks were employed to create steps for thickness measurements. Due to the layers being much thinner than the substrates, stress (σ) calculations utilized the modified Stoney Equation (1) for thin layers, considering the biaxial nature of stress rather than uniaxial [25,26,27].

(1) $$\sigma =\frac{M_{s}t_{s}^{2}}{6t_{l}}\left(\frac{1}{R}-\frac{1}{R_{0}}\right),$$

where M_s_, t_s_, t_l_, R, and R_0_ are the biaxial modulus of substrate, the substrate thickness, the layer thickness, the radii of the curvatures of the substrate after and before the deposition, respectively. The negative stress value indicates compressive stress, resulting in a convex surface, while a positive stress value signifies tensile stress, leading to a concave surface.

#### 2.2.2. Raman Spectroscopy

Raman spectroscopy measurements were conducted using a Renishaw inVia Raman microscope (Reinshaw plc, Gloucestershire, UK) with a 532 nm laser and a 2400 L/mm grating. The laser power was set to 0.1 mW, and the acquisition time was 30 s. With these settings, the diameter of the focal spot was approximately 3 µm. The measured area was carefully selected to avoid inclusions, cracks, and other defects in the field of view. However, when the density of inclusions was high, it was unavoidable to include some inclusions in the field of view, as observed in the samples investigated in Section 3.3: Dark Current Analysis. 

#### 2.2.3. Calculation of Thermal Expansion Coefficient

In order to determine the coefficient of thermal expansion (CTE) and elastic modulus of a-PbO, we paired substrates with diverse thermomechanical properties and co-deposited a-PbO on them. Four pairs were prepared for this study: three were identical (with the same substrates and deposition settings) to assess repeatability; one pair utilized different substrates with deposition conditions similar to the first three pairs for assessing reliability and generality of the calculation method (Table 2).

By conducting the aforementioned curvature measurements on the pairs as a function of temperature, CTE and elastic modulus can be calculated using Equation (2) for each sample of the couples [28,29,30].

(2) $$m=\left(\frac{6t_{l}}{t_{s}^{2}}\right)\left(\frac{M_{l}}{M_{s}}\right)\left(\alpha_{s}-\alpha_{l}\right)$$

where *m* is the slope of the curvature–temperature curves. The subscripts *l* and *s* refer to the layer and the substrate, respectively.

Curvature measurements were conducted at temperatures of 25° C, 50° C, and 75° C using a Peltier element powered by a current supply (BK Precision 1900, B&K Precision Corporation, Yorba Linda, CA, USA). The current supply was controlled via an Arduino board, which incorporated a PID controller coded in the LabVIEW 2020 (National Instruments, Austin, TX, USA) environment. Temperature was measured using an active thermistor integrated circuit (MCP9700B, Microchip Technology, Chandler, AZ, USA), which was attached to the sample holder near the detector. After setting a new temperature value, a 10 min waiting period was allowed for temperature stabilization. The results were plotted, and linear fits were applied to the curvature-temperature curves to determine the slopes.

#### 2.2.4. Dark Current Measurement

To investigate the influence of layer structural inhomogeneity on the dark current as a function of bias voltage, we employed procedures detailed in Ref. [8]. The samples were placed in an aluminum box to prevent photogeneration. Using a Standford Research Systems PS350 power supply (Stanford Research Systems, Sunnyvale, CA, USA), a purpose-developed software, developed by our group, applied a pre-programmed positive bias voltage series to the ITO bottom electrode. The dark current, measured back from the Au top electrode, was recorded with a Keithley 35617EBS electrometer (Keithley Instruments, Solon, OH, USA). A 30 s delay was implemented between applying the bias and reading out the signal. Prior to the measurement, the top and bottom electrodes were short-circuited for 30 min to ensure the detrapping of charge carriers.

## 3. Results

### 3.1. Stress and Surface Roughness Analysis

**Figure 3:**
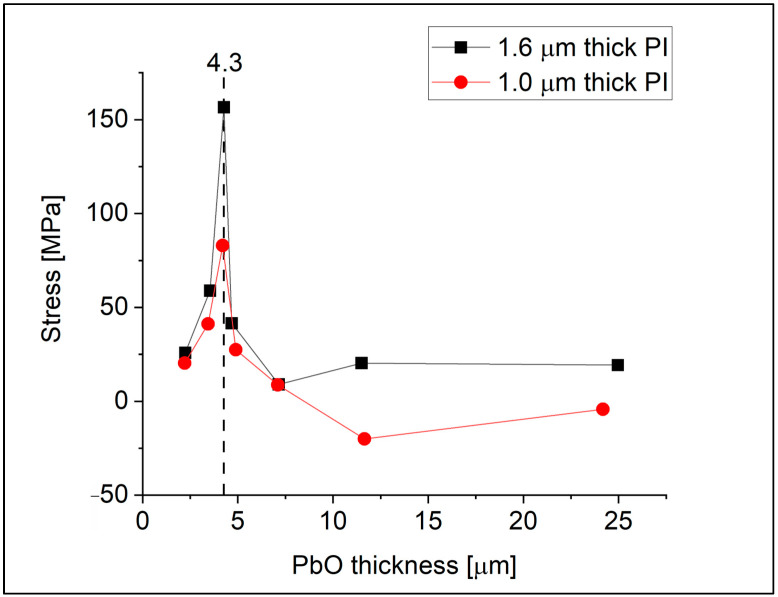
Residual stress in a-PbO layers as a function of a-PbO thickness.

In this study, a series of a-PbO layers of varying thicknesses was deposited on boro-aluminosilicate glass substrates covered with polyimide (PI) layers. The mechanical stress of the layers was calculated, and Figure 3 illustrates the stress as a function of the a-PbO layer thickness. The samples were fabricated in pairs in the same deposition process. In each pair, one sample was covered with a 1.0 µm-thick layer, while the other had a 1.6 µm-thick PI layer. The stress exhibits a rapid increase until approximately 4–5 µm layer thickness, followed by a sudden drop. Two sample types with different PI thicknesses show a similar trend, indicating stress release. Optical microscopy revealed that stress release is accompanied by cracking in the a-PbO layer.

**Figure 4:**
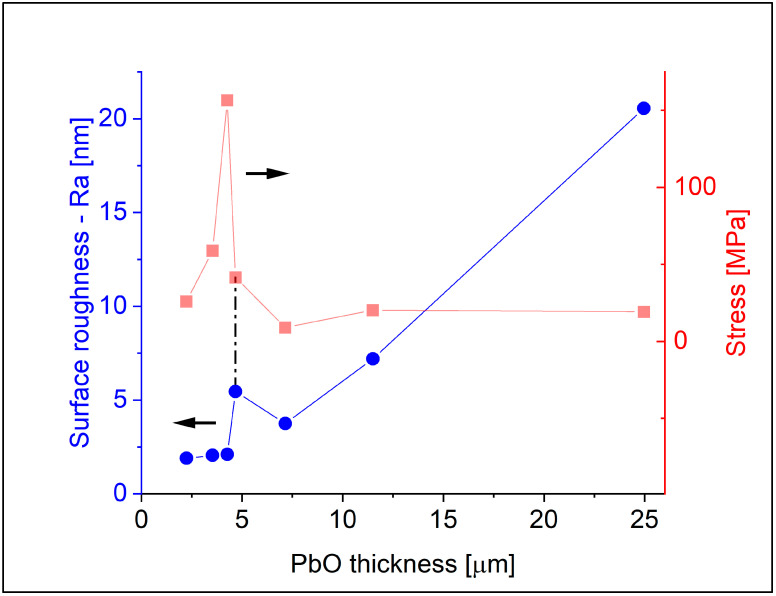
Surface roughness (Ra) as a function of PbO thickness measured on samples with PI thickness 1.6 µm (blue curve). For comparison, the residual stress data from Figure 3 are also shown (red curve).

For quantitative information on crack distribution over a larger area, we employed a profilometer to measure and calculate the surface roughness of the samples. Figure 4 illustrates the surface roughness in terms of Ra (average roughness) measured on the same 1.6 µm-thick PI samples used for stress measurements and correlates it with stress measurements. For thin layers (thinner than ~5 µm), the roughness value remains relatively low and does not change significantly up to the critical stress value, where Ra increases due to crack formation. Beyond this point, the roughness exhibits an increasing tendency with thickness as more cracks are formed. Roughness measurements for the samples with the thinner polymer showed a similar trend. It is essential to note that this method is not suitable for examining the dimensions of cracks due to the tip radius of the stylus being in the same range as the width of the cracks.

### 3.2. Coefficient of Thermal Expansion and Elastic Modulus

**Figure 5:**
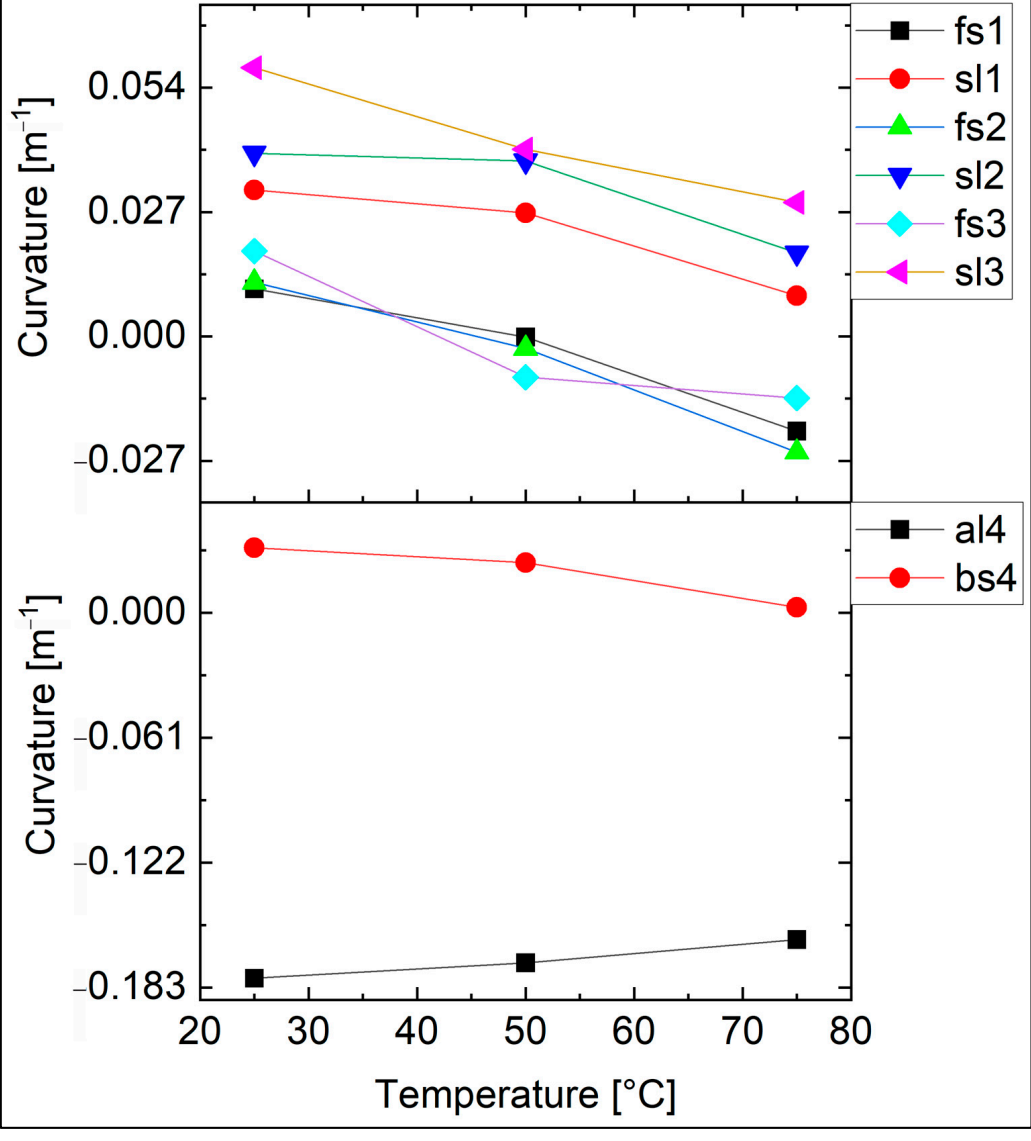
Curvature of the samples as a function of temperature for four identical co-deposited pairs. The abbreviations refer to the substrate type (fs—fused silica, sl—sodalime, bs—boro-aluminosilicate, and al—aluminium) while the numbers indicate the pairs. The thermal expansion coefficient and the elastic modulus of PbO were calculated using the slopes of these curves.

Figure 5 depicts the curvature of samples from pairs with a thicker a-PbO layer. As the curves exhibit a roughly linear trend, we employed linear fitting to calculate slopes for Equation (2). To ensure accuracy and assess the repeatability of our calculation approach, we used three identical pairs of samples for fused silica (fs) and soda-lime glass (sl) substrates, and one pair consisting of different substrates—aluminum (al) and boro-aluminosilicate glass (bs). These sample pairs were labeled by group number (e.g., fs1, fs2, fs3) and analyzed independently to verify consistency in the calculated CTE values. The properties of both substrates and thin layers, along with the calculated thermal expansion coefficient of a-PbO, are presented in Table 2. The average of the calculated values, representing the thermal expansion coefficient of amorphous lead oxide, is 13 × 10^−6^ K^−1^. Additionally, the average calculated elastic modulus is 330 GPa.

### 3.3. Dark Current Analyses

**Figure 6:**
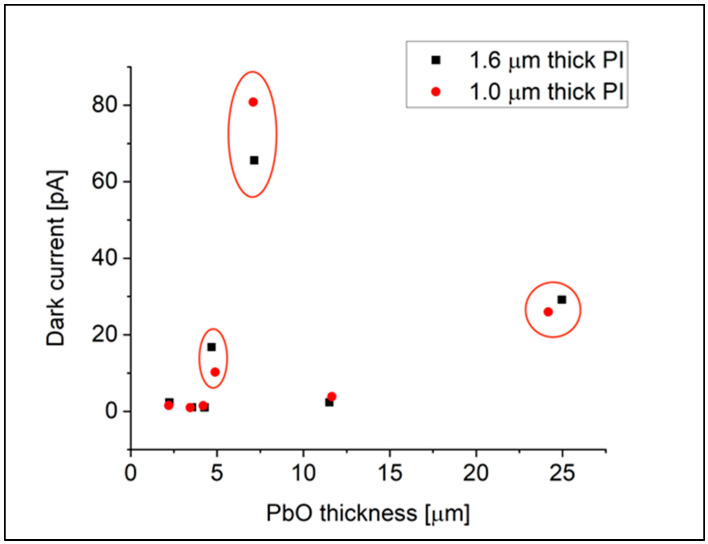
Dark current as a function of a-PbO thickness at 10 V/µm bias voltage.

Figure 6 presents the dark current measurements for the same set of samples used in the stress analysis. All samples were deposited on boro-aluminosilicate glass substrates, with the a-PbO layer thickness varying from 4 to 25 μm. The samples were fabricated in pairs during the same deposition process to ensure consistency. In each pair, one sample was covered with a 1.0 μm PI layer and the other with a 1.6 μm PI layer. Notably, for the first two circled pairs in the figure, the crucible temperature reached 1070 °C during deposition. For the thickest sample, the deposition time was extended to 150 min.

**Figure 7:**
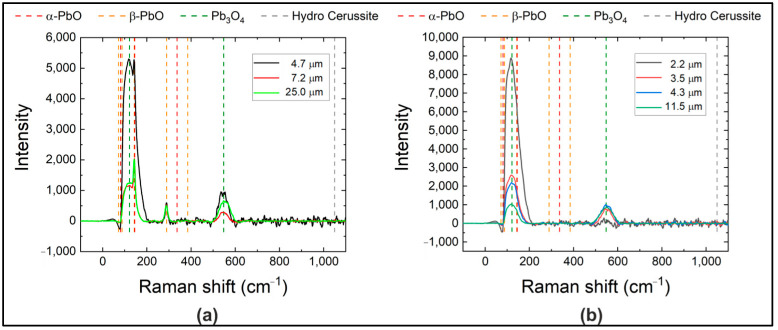
Raman spectra of samples with thicker polymer from circled (**a**) and uncircled (**b**) pairs from Figure 6.

As seen in Figure 6, layers with thicknesses less than 5 µm are characterized by a low dark current that does not exceed 2.5 pA/mm^2^. This low dark current magnitude correlates well with the structural homogeneity of these samples, as reflected in Raman measurements provided in Figure 7b—as can be seen, the Raman spectrum for thin layers is dominated by a broad band with a maximum near 120 cm^−1^, typical for an amorphous phase. Almost the same low dark current and Raman spectra were observed in a thicker sample with an a-PbO layer thickness of 11.5 µm. We attribute the preserved structural homogeneity in this sample to the crucible temperature not exceeding 1070 °C during a-PbO deposition and a relatively short deposition time.

For the rest of the samples, dark current values (highlighted with circles in Figure 6) were much higher. The characteristic peaks of β-PbO (orthorhombic phase) observed in the Raman spectra, as shown in Figure 7a, provide evidence for the presence of polycrystalline inclusions in these layers. In two of them with comparatively thin a-PbO layers of 4.7 µm and 7.2 µm, the appearance of polycrystalline inclusions is attributed to elevated deposition temperatures (exceeding approximately 1070 °C of the crucible), even for a short duration (a few minutes), leading to the formation of numerous crystallographic defects and crystallite inclusions within the layer. The heightened inclusion density subsequently results in significantly elevated dark current. An elevated dark current was also observed in samples with prolonged deposition times (larger layer thickness of 25 µm) despite maintaining the crucible temperature below 1070 °C. The Raman spectra in Figure 7a demonstrate a correlation between the elevated dark current level and the presence of the characteristic peaks of β-PbO in this sample.

Table 3 summarizes the observed correlations between a-PbO layer thickness, stress, surface roughness, dark current, and crystallinity.

## 4. Discussion

**Figure 8:**
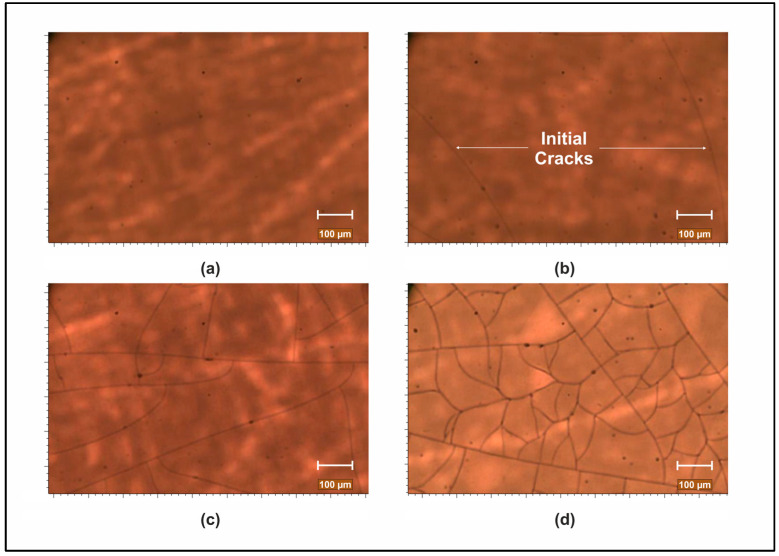
Stress development with the growing layer illustrated through samples with thicknesses around the critical value (**a**–**c**) and one sample with a larger thickness (**d**). The thicknesses are 3.5 µm (**a**), 4.3 µm (**b**), 4.7 µm (**c**), and 11.5 µm (**d**).

The observed dependence of stress on a-PbO layer thickness in Figure 3, where stress suddenly drops after reaching a critical layer thickness (or rather a critical initial stress), is attributed to crack formation. Samples below this threshold of ~4–5 µm exhibit minimal cracking, while those exceeding it experience a sharp rise in crack density (Figure 8). The correlation between stress and cracks supports the notion that stress release occurs through crack propagation.

**Figure 9:**
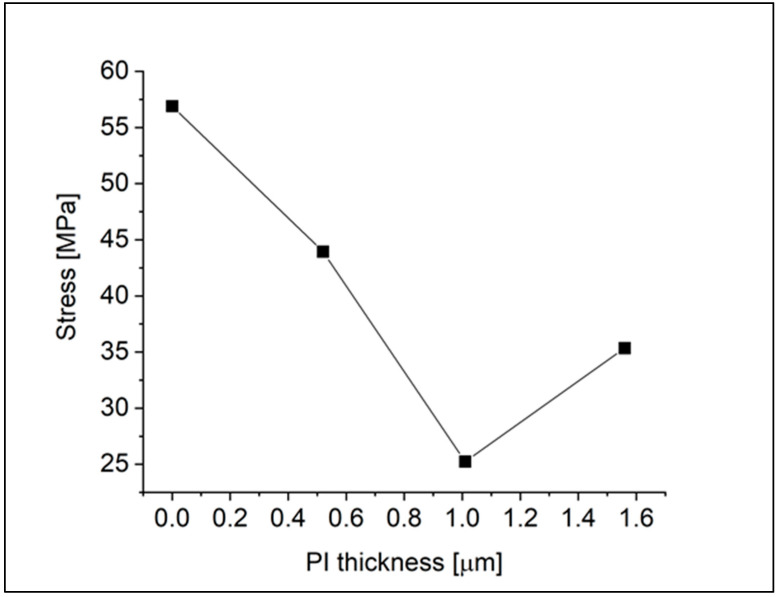
Residual stress in a-PbO layers as a function of PI thickness.

To deepen our understanding of how the detector structure can influence stress and crack propagation, we also discuss the impact of PI thickness on stress reduction. Figure 9 shows the residual stress measured in a series of detector prototypes, all with an average a-PbO layer thickness of 3.8 µm, but with varying PI layer thicknesses. All a-PbO layers were co-deposited in the same process to ensure that any observed differences in stress are attributable solely to the PI thickness, thereby excluding other variables such as deposition conditions or substrate effects. For three samples, the PI thickness was adjusted with the speed of the spin-coater, and one sample had no PI at all. The results show that the polymer can reduce intrinsic stress in the PbO layers up to a certain threshold. Indeed, a soft polymer like PI mitigates the shear strain originating from the layer’s adhesion to its substrate [11]. Given that shear strain is known to drive faster lateral growth of the amorphous matrix, when introducing tensile strain, it is reasonable to assume that its suppression reduces the intrinsic stress. However, in structures with thicker blocking layers, the stress level increases. We attribute this increase to differences in thermal expansion coefficients, where the impact of these differences takes over the stress reduction achieved through shear strain relief. Indeed, the CTE of the PI2610 we use is significantly smaller (3 × 10^−6^ K^−1^) than that of a-PbO, as demonstrated in Section 3.2. Figure 9 also illustrates the same trend; regardless of a-PbO layer thickness, thicker PI layers (1.6 µm) result in larger measured residual stress.

**Figure 10:**
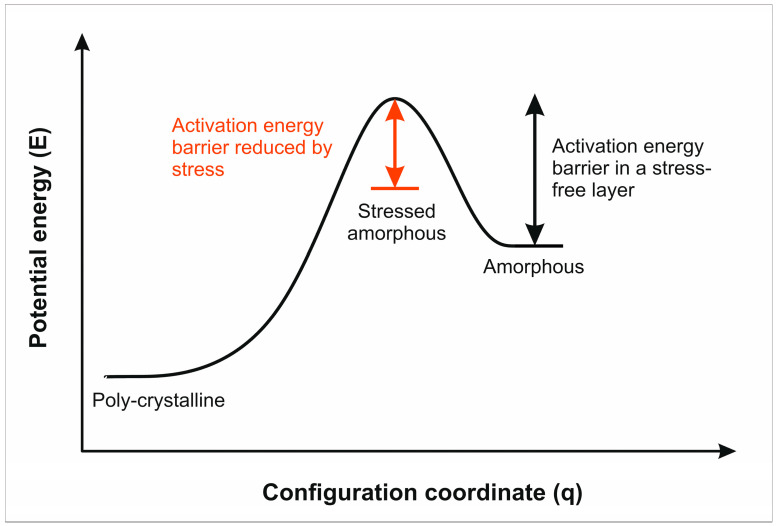
Energy–configuration diagram for the structural transformation in a-PbO layers and the effect of stress on activation energy barrier.

Although driven by stress in the growing layers, crystallization is a thermally activated process and is expected to accelerate at elevated temperatures. It is plausible to assume that, similar to other materials with different polymorphs, the ground state configurations of thermodynamically stable poly-PbO and metastable a-PbO forms are separated by a potential barrier that prevents structural transformation toward a fundamentally more stable crystallographic configuration. However, the combination of direct heating from the crucible and the ion source may lead to locally accumulating temperature in the poorly thermally conductive growing layer, thus facilitating local re-crystallization during the deposition process. Additionally, similar to a-Se, local strain and stress may promote re-crystallization, suggesting that the height of the potential barrier is altered by the presence of stress and decreases with the accumulation of stress in the grown layers. Therefore, stress increases the probability of forming crystallographic defects and crystalline inclusions in the growing layer. Figure 10 presents a schematic representation of the effect of initial stress on the activation energy barrier associated with the amorphous-to-polycrystalline transition.

Our experiments revealed that the presence of initial stress in the grown layers of a-PbO poses a significant obstacle. This stress leads to the formation of structural inclusions that significantly impact the performance of a-PbO detectors, particularly in relation to dark current. On the other hand, cracks, formed to relieve stress, create technological challenges in depositing a-PbO layers with the required thickness. Layers exceeding ~30 µm tended to delaminate from the substrate. Our measurements corroborate earlier suggestions that stress and thermal effects can induce defects in amorphous semiconductors, and we provide new results for a-PbO, including the determination of the critical thickness for crack formation and the thermal expansion coefficient. Our results underscore the critical need to address stress-related structural modifications in the deposition process to achieve thicker a-PbO layers suitable for medical imaging applications. Both structural defects, i.e., inclusions and cracks, can be prevented by eliminating the build-up of stress in grown layers.

As stress results from a mismatch in CTE between the substrate and the grown layer, a plausible solution seems to be using a substrate with a CTE similar to that of the grown layer or tuning a deposition technique for room temperature deposition. Although our focus in this study was on a-PbO, our findings may be applicable to other amorphous x-ray photoconductors and blocking structures.

## 5. Conclusions

In this study, we investigated the evolution of stress and the formation of cracks and crystalline inclusions in a-PbO layers, addressing challenges associated with increasing layer thickness essential for medical imaging purposes. Particularly, we found that the formation of structural inclusions and cracks in thick a-PbO layers can be understood by a stress-induced crystallization model that accounts for the evolution of intrinsic stress with the thickness of a-PbO samples: we observe that tensile stress initially rises with layer thickness and is subsequently relieved by the occurrence of cracks. As the primary source of intrinsic stress arises from the thermomechanical mismatch between the photoconductor layer and the glass-blocking layer commonly used in photodetector devices, we calculate the thermal expansion coefficient and elastic modulus of a-PbO.

The heat accumulation originating from the furnace and the ion source triggers stress-induced crystallization, resulting in the formation of crystalline inclusions in the growing layer. These inclusions not only increase dark current but also accelerate crack propagation. Once the crystallographic inclusions have emerged, they are permanent, as they are associated with the thermodynamically stable phase of β-PbO. They not only expedite crack propagation but also markedly increase device dark current, despite the typically low dark current advantage of a-PbO layers.

In summary, this study elucidates the interplay between stress, crystallization, and performance degradation in thick a-PbO layers. The challenges identified highlight the necessity of (1) substrate and blocking layer materials with matched thermal expansion coefficients to mitigate thermomechanical stress, and (2) optimized deposition parameters, such as crucible temperature, ion energy, and deposition time, to minimize heat accumulation and subsequent crystallization. Addressing these factors is critical for producing detector-grade a-PbO layers with a low dark current and structural integrity.

## Figures and Tables

**Table 1:** Summary of substrate types and their properties.

Substrate Type	Thickness (µm)	Elastic Modulus (GPa)	CTE (10^−6^ K^−1^)	Coating
Aluminum (al)	820	102.8	23.40	--
Soda-lime (sl)	700–750	91.3	9.00	ITO
Boro-aluminosilicate (bs)	780	67.0	3.30	ITO
Fused silica (fs)	1150–1210	88.1	0.55	ITO

**Table 2:** CTE of a-PbO layers. The substrate and layer thicknesses, substrate CTE, and elastic modulus are also indicated.

Pairs	Substrate Thickness (t_s_) µm	Layer Thickness (t_l_) µm	Substrate Elastic Modulus (D_s_) GPa	CTE of the Substrate (α_s_) 10^−6^ K^−1^	Calculated CTE of a-PbO (α_PbO_) 10^−6^ K^−1^
fs1	1170	4.1	88.1	0.55	11.7
sl1	760	4.1	91.3	9.00	
fs2	1210	4.3	88.1	0.55	13.7
sl2	750	4.2	91.3	9.00	
fs3	1150	4.1	88.1	0.55	13.1
sl3	700	4.1	91.3	9.00	
al4	820	4.0	102.8	23.40	12.8
bs4	780	4.1	67.0	3.30	

**Table 3:** Summary of the relationship between a-PbO layer thicknesses, intrinsic stress, measured surface roughness and dark current density, and observed crystallinity.

a-PbO Thickness (µm)	Stress (MPa)	Surface Roughness (nm)	Dark Current (pA)	Crystallinity
2.2	26	1.9	2.4	Low
3.5	59	2.1	1.1	Low
4.3	157	2.1	1.0	Low
4.7	42	5.5	16.8	High
7.2	9	3.8	65.6	High
11.5	20	7.2	2.4	Low
25.0	19	20.6	29.2	High

## Data Availability

The original contributions presented in this study are included in the article. Further inquiries can be directed to the corresponding author.

## Abbreviations

The following abbreviations are used in this manuscript:

a-PbO	Amorphous Lead Oxide
a-Se	Amorphous Selenium
FPXI	Flat-Panel X-ray Imager
a-Si:H	Hydrogenated Amorphous Silicon
TFT	Thin Film Transistor
HARP	High-gain Avalanche Rushing Photoconductor
SEM	Scanning electron microscopy
ITO	Indium-tin-oxide
PI	Polyimide
al	Aluminum
sl	Soda-lime
bs	Boro-aluminosilicate
fs	Fused silica
CTE	Coefficient of Thermal Expansion
